# Editorial: Neuroendocrine research in health and disease, volume II

**DOI:** 10.3389/fnins.2023.1253725

**Published:** 2023-08-14

**Authors:** Yu-Feng Wang, Keith Maurice Kendrick, Xue Qun Chen, Lei Sha

**Affiliations:** ^1^International Translational Neuroscience Research Institute, Zhejiang Chinese Medical University, Hangzhou, China; ^2^MOE Key Laboratory for Neuroinformation, The Clinical Hospital of Chengdu Brain Science Institute, University of Electronic Science and Technology of China, Chengdu, China; ^3^Department of Neurology of Second Affiliated Hospital and School of Brain Science and Brain Medicine, Zhejiang University, Hangzhou, Zhejiang, China; ^4^Department of Neuroendocrine Pharmacology, School of Pharmacy, China Medical University, Shenyang, China

**Keywords:** endocrine, hormone, hypothalamus, neuropeptide, pituitary

In this 2nd volume of “Neuroendocrine Research in Health and Disease”, we collected papers of neuroendocrine research in the following themes.

## 1. Mental health and behaviors

Mental health and behaviors are closely associated with balanced actions of hormones that are under the control of neuroendocrine activity (Prencipe et al., 2023). Some patients present extremely difficult and challenging behaviors due to brief stressors in life or changes in neural structures in association with neuroendocrine disorders involving oxytocin (Le et al., 2022), arginine vasopressin (Parker et al., 2019), and corticosteroids (Chen, 2022). In this topic, Li et al. presented that individuals with obsessive-compulsive disorder have biomarkers like docosapentaenoic acid and 5-hydroxytryptophan, which help obsessive-compulsive disorder identification and prediction of sertraline treatment outcome, respectively.

## 2. Neurodegenerative disorders

Neurodegenerative diseases are caused by loss of functions or death of cells in the brain and spinal cord because of genetic mutation, alcoholism, tumor, traumatic injuries, toxins, chemicals, and viruses. The diseases frequently get worse over time and lack efficient therapy (Amanollahi et al., 2023). Sex steroids have been shown to play a role in the progression of specific forms of neurodegenerative diseases (Saleh et al., 2023; Terrin et al., 2023). In this topic, Hu et al. further explored the mechanisms underlying estrogen reduction-associated higher incidence of Alzheimer's disease in women than in men during aging. That is, the higher prevalence of Alzheimer's disease in women is related to changes in brain levels of estrogen that reduce β-amyloid levels, possibly by increasing early growth response-1-stimulated acetylcholinesterase expression.

## 3. Seizures

Seizure can result from a genetic disorder or an acquired brain injury that are usually localized and from high fevers, alcohol or drug withdrawal, and low blood sugar that affect whole body. When some neurons become excited abnormally, they cause excitation of neurons in their adjacent regions or associated neural networks (Reddy, 2020). In this topic, He et al. reported that pilocarpine-induced seizures can selectively activate the hypothalamic orexinergic but not melanin-concentrating hormone neurons in rats with sleep disorders, which involves the spread of epileptic activity from amygdala to the hypothalamus, thereby contributing to the disturbance of sleep-wakefulness in temporal lobe epilepsy.

## 4. Neural regulation of neuroendocrine activity

Neuroendocrine activity is regulated by neural and humoral factors, including autonomic nerve activity (Besecker et al., 2018; Breit et al., 2018; Bonaz, 2022). For example, both intrapancreatic ganglia and extrapancreatic nerves have an important influence on pancreatic endocrine function (Li et al., 2019). In this topic, Zhu et al. further explored that stimulating the cutaneous receptive field formed by the auricular branch of the vagal nerve in the outer ear helps alleviate maladaptive neural plasticity underlying the pathology of several pediatric neurodevelopmental and psychiatric disorders, such as autism spectrum disorder, attention deficit hyperactivity disorder, disruptive behavioral disorder, and stress-related disorder. Thus, this technique provides a promising alternative treatment for pediatric disorders which do not respond to other interventions.

## 5. Therapeutic usage in endocrine diseases

Many endocrine diseases can be treated with hormones. Hormone replacement therapy (HRT) in aged women has a long history to improve menopausal symptoms while many other health issues like Alzheimer's dementia (Saleh et al., 2023) can also be relieved by HRT. In this topic, Kawahara et al. reported the application of HRT in patients with generalized anhidrosis caused by hypothalamic pituitary germinoma. Patients with anhidrosis were diagnosed with germinoma based on magnetic resonance imaging and pituitary biopsy findings, in which panhypopituitarism was identified. Desmopressin, a synthetic analog of arginine vasopressin, resolved the dehydration symptoms, the efficiency of which is associated with the extent of hypothalamic involvement (Kawahara et al.). The study on this rare sweating disease provides novel pathological mechanism underlying the “diabetes insipidus” and its optimal therapy.

## 6. Anti-microbial infection

Many studies have revealed that neuropeptides have antimicrobial properties (Aresti Sanz and El Aidy, 2019; Pascal et al., 2022). For example, arginine vasopressin can decrease sepsis-induced pulmonary inflammation, and modulate stress responses by changing cytokine expression and release from immune cells (Russell and Walley, 2010). Oxytocin antagonism promotes replication and translation of hepatitis C virus (Zhu et al., 2017). In this topic, the antimicrobial capacity of hormones is further explored in the treatment and prevention of COVID-19. It is known that COVID-19 caused by severe acute respiratory syndrome coronavirus 2 and that many hormones have been identified as potential agents for treating COVID-19 diseases. Among them, oxytocin possibly inactivates the spike protein of this virus and blocks viral entry into cells via angiotensin-converting enzyme 2 through multiple approaches. In addition, oxytocin can also reduce viral density on the surface of body by increasing parasympathetic outflow and the secretion of body fluids (Wang et al.). This review provides medical workers a new strategy dealing with COVID-19 and other infectious diseases.

## 7. Conclusion and perspectives

As a whole, neuroendocrine research provides novel view of neuroendocrine regulation of social and non-social behaviors, cognition and a variety of body functions by hormonal actions via endocrine, autocrine, paracrine and intracrine routes. Thus, they play dominant roles in shaping normal life activity. By contrast, abnormal expression and malfunction of these neuropeptides underlie many diseases including but not limited to stress, hyperthyroidism, infertility, hypertension, hyponatremia, obesity, diabetes mellitus, breastfeeding failure, intellectual disability, psychiatric disorders, metabolic disorders as well as increased susceptibility to tumors and infectious diseases.

Future directions in neuroendocrine studies may include analyzing functions and regulation of classical neuropeptides and hormones, identifying and characterizing novel neuropeptides, exploring cellular and molecular mechanisms underlying neuropeptide actions, constructing the integration center for instinctive behaviors, establishing microbe-gut-brain axis. Certainly, application of modern biomedical techniques such as magnetic resonance imaging (Chen et al., 2023), whole-brain mapping of neuropeptide projections (Zhang et al., 2021; Lin et al., 2023), optogenetics (Perkinson et al., 2021; Pan et al., 2022), in neuroendocrine research is also highly expected.

## Author contributions

Y-FW: Conceptualization, Resources, Validation, Writing—first draft and editing. KK: Conceptualization, Resources, Validation, Writing—review and editing. XC: Conceptualization, Resources, Validation, Writing—review and editing. LS: Conceptualization, Resources, Validation, Writing—review and editing.
